# Mesenchymal Stem Cell–Derived Exosomes: A Promising Biological Tool in Nanomedicine

**DOI:** 10.3389/fphar.2020.590470

**Published:** 2021-01-25

**Authors:** Wumei Wei, Qiang Ao, Xiaohong Wang, Yue Cao, Yanying Liu, Song Guo Zheng, Xiaohong Tian

**Affiliations:** ^1^ Department of Tissue Engineering, School of Fundamental Science, China Medical University, Shenyang, China; ^2^ Institute of Regulatory Science for Medical Device, National Engineering Research Center for Biomaterials, Sichuan University, Chengdu, China; ^3^ Department of Rheumatology and Immunology, Peking University People’s Hospital, Beijing, China; ^4^ Department of Internal Medicine, The Ohio State University College of Medicine and Wexner Medical Center, Columbus, OH, United States

**Keywords:** Nanomedicine1, mesenchymal stem Cells2, exosomes3, mesenchymal stem cell-derived exosomes4, extracellular vesicles5

## Abstract

As nano-scale biological vesicles, extracellular vesicles (EVs)/exosomes, in particular, exosomes derived from mesenchymal stem cells (MSC-exosomes), have been studied in the diagnosis, prevention, and treatment of many diseases. In addition, through the combination of nanotechnology and biotechnology, exosomes have emerged as innovative tools for the development of nanomedicine. This review focuses on a profound summarization of MSC-exosomes as a powerful tool in bionanomedicine. It systemically summarizes the role of MSC-exosomes as a nanocarrier, drug loading and tissue engineering, and their potential contribution in a series of diseases as well as the advantages of exosomes over stem cells and synthetic nanoparticles and potential disadvantages. The in-depth understanding of the functions and mechanisms of exosomes provides insights into the basic research and clinical transformation in the field of nanomedicine.

## Introduction

Recently, there has been growing concern about the development of exosomes in response to the increasing demands of human health. Exosomes, as natural nano-scale particles, have various advantages in comparison with other engineered nanoparticles. In the last decade, basic research and clinical trials related to exosomes have penetrated many fields of medicine (Bebelman et al., 2020; Fang et al., 2020; Kalluri and LeBleu, 2020; Moller and Lobb, 2020). Because exosomes can be secreted by almost all cells and different types of exosomes can perform a variety of functions, how to choose the ideal cells for research is a question that needs to be considered. Mesenchymal stem cells (MSCs) are an important branch of stem cells and have been considered as promising seed cells in the research on regenerative medicine, cell therapy, and tissue engineering (Zhao et al., 2018b; Cui et al., 2018; Riazifar et al., 2019). Accordingly, research based on exosomes derived from MSCs (MSC-exosomes) has great value.

MSC-exosomes not only have the advantages of exosomes, but also the characteristics of MSCs. Numerous studies have shown that they are therapeutic in a variety of diseases, including tumors (Zhao et al., 2018b), neurodegenerative diseases (Riazifar et al., 2019), cardiovascular (Cui et al., 2018) and cerebrovascular (Moon et al. 2019) diseases, wound repair (Rao et al. 2019), and so on. In this aspect, MSC-exosomes can be regarded as potential nano-therapeutic agents. Furthermore, as natural nano-drug delivery vehicles, MSC-exosomes combined with engineering technology (Chen et al., 2019a; Zhuang et al., 2019) have achieved promising results in disease treatment according to current research. Consequently, improvement in our understanding of the mechanisms, research progress, and applications underlying MSC-exosomes provides new insight into potential therapeutic strategies for nanomedicine.

The remainder of this paper is organized as follows. In Section *Exosomes*, we briefly describe the relationship between exosomes and nanomedicine. Simultaneously, we briefly mention some examples of exosomes in basic research and clinical applications for disease diagnosis and treatment. Section *MSC-Exosomes* introduces MSCs, MSC-exosomes, and their pros and cons. Subsequently, the updated research and application of MSC-exosomes are presented from two perspectives. One is as a nanocarrier used for drug delivery, and the other is as nanomedicine (nano-drug) used for treatment. Section *Conclusion and Outlook* draws short conclusions and offers an outlook.

## Exosomes

Exosomes are extracellular vesicles (EVs) with a diameter of 30–150 nm formed by fusion of multivesicular cell membranes (Gurunathan et al., 2019). EVs are mainly divided into exosomes, microvesicles, and apoptotic bodies according to their size, biological characteristics, and formation processes. They can be released by almost all cells, can be transferred to target cells through cell-to-cell communication, and perform a variety of biological functions. The current progress on exosomes in the diagnosis and treatment of diseases provides an important basis for their future application in medicine (Li et al., 2019).

### Exosomes and Nanomedicine

**FIGURE 1:**
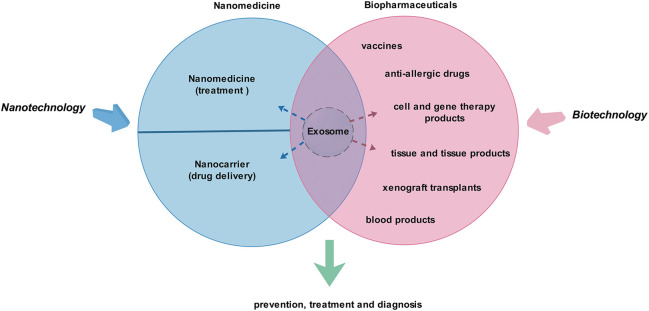
Exosomes are promising “bio-nanomedicine.” Nanotechnology and biotechnology are closely related to nanomedicine and biopharmaceuticals. Exosomes share the common characteristics of biopharmaceuticals and nanomedicines, so they are natural “bio-nanomedicine.” They may play a role in the prevention, treatment, and diagnosis of diseases.

As biologically active vesicles, exosomes have the characteristics of biopharmaceuticals and nanomedicine (nano-drugs). Therefore, exosomes are natural “bio-nanomedicine” (Figure 1). Of course, strictly speaking, there is still a big gap between exosomes and biopharmaceuticals (Farley and Link, 2009; Gils et al., 2017; Dreesen et al., 2018) or nano-drugs (Kim et al., 2010; Lammers et al., 2011; Zhao, 2018), so they are not yet clearly defined. A number of studies show that exosomes play therapeutic, diagnostic, and other roles through their functional proteins and nucleic acids. Various types of artificial modification can also be used to achieve the corresponding therapeutic or drug-loading purposes, and these modifications can be achieved through biotechnology or/and nanotechnology, etc. In view of the complexity of production and application of biological agents, there is still a long way to go for exosomes to achieve clinical transformation.

With the rapid progress of nanomedicine and technology integration, exosomes/EVs combined with biotechnology and nanotechnology have novel applications. At present, the major directions for study of exosomes/EVs are concentrated in three parts, including mediating cell behavior through intercellular communication, the screening of biomarkers, and the researching and developing of drug carriers.

Given exosomes’ desired functions, they can be better applied in the fields of medicine and health. For example, some studies report that proteomics and biophysical techniques can be used to quantify proteins related to exosomes/EVs at the molecular level, thereby promoting the research process of exosome/EV engineering (Corso et al., 2019). In addition, there are also some artificial exosomal manufacturing techniques, such as extruding cells through nanopores and other methods to make exosome-like vesicles (Colao et al., 2018).

**FIGURE 2:**
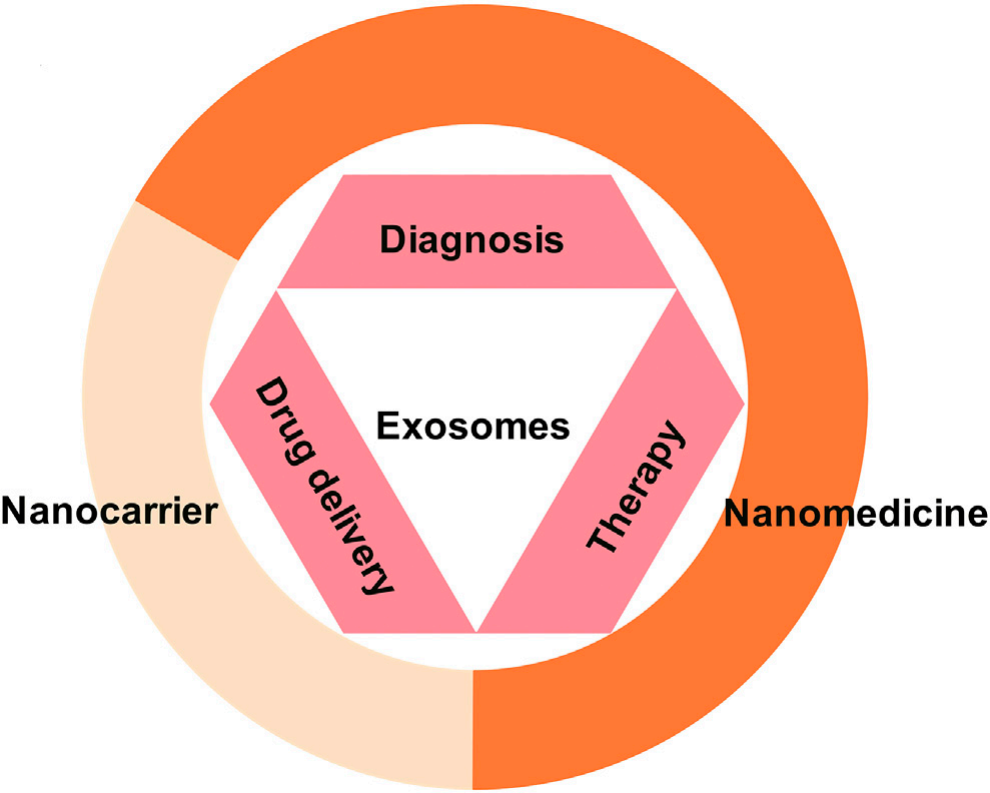
Exosomes as nanomedicine. As a natural nanoparticle, exosomes play an important role in the diagnosis, treatment, and prevention of diseases and have become a promising clinical nanomedicine. At present, exosome products have been used for disease diagnosis. In addition, basic and clinical research on exosomes for the treatment of diseases and drug delivery is also under intense development.

So far, our understanding of exosomes/EVs used for diagnosis has matured (Liu et al., 2019a; Fan et al., 2019b; Brittain et al., 2019; Zhu et al., 2020). It can be achieved by analyzing the characteristics of exosomes/EVs isolated from body fluids to diagnose underlying diseases (Pang et al., 2019). In addition to diagnosis, exosomes/EVs are also being studied as new therapy (Wang et al., 2019b; Majer et al., 2019; Otero-Ortega et al., 2019; Rodrigues et al., 2019; Xie et al., 2019). However, most of these studies are in the preclinical stage and are rarely tested in the clinic. Early work shows that exosomes play a significant role in tumors (Rodrigues et al., 2019), autoimmune diseases (Xie et al., 2019), and so on. In summary, the basic research on exosomes/EVs is divided into two aspects. On one hand, they can be used as drug delivery carriers for cell-targeting therapy. On the other hand, specific exosomes/EVs from different sources can be used directly or indirectly for multiple kinds of diseases (Pang et al., 2019) (Figure 2).

### Clinical Application and Commercial Production of Exosomes/EVs

With the increasing popularity of exosome/EV research, many booming commercial companies have emerged, and some established, large-scale, biopharmaceutical companies have also begun to invest in the development and production of exosome/EV products (Table 1). Exosome/EV products have made their debut in the field of diagnosis. The world’s first exosome-based diagnostic kit, ExoDX Lung (ALK), developed by Exosomes Diagnostics, passed FDA certification in 2016. Although there are no products that have been formally applied to the clinic in terms of therapeutic applications, many products are already at the stage of preclinical research or in clinical trials and have achieved certain results (Ibrahim et al., 2014; Webb et al., 2018).

**TABLE 1:** Major commercial companies researching and producing exosome products.

Company	Product/Research
Aethlon medical	Development of exosomal-based candidate products for the diagnosis and management of neurological disorders and cancer
Anjarium biosciences	Focus on therapeutic RNA cargo, both long as well as short RNAs, including those that are chemically optimized for very high potency
Aruna biomedical	AB126, neuro exosomes used across the blood–brain barrier to treat stroke
Biocept	Focus on developing innovative diagnostic solutions all from a simple blood test, known as a liquid biopsy
Capricor therapeutics	CAP-2003, exosomes produced by cardiosphere-derived cells
Ciloa	Delivery of membrane proteins
Codiak biosciences	Exosomal targeted delivery of sting agonist or IL-12
Creative medical technology holdings	Focused on regenerative medical solutions for unmet urological, neurological and orthopedic needs
ExananoRNA	Committed to promoting the transfer of biological nanotechnology, especially RNA nanotechnology, from laboratory research to clinical applications
ExoCoBio	A pharmaceutical R and D and cosmeceutical company. In the cosmeceutical business, ExoCoBio has launched its own exosome-based brands and products, such as EXOMAGETM, CelltweetTM, and ASCE + TM.
Exosome diagnostics	Develop novel and sensitive diagnostic methods based on exosomes, such as prostate, bladder, kidney, breast, glioblastoma, and other cancers
Exovita biosciences	Breast cancer treatment
Evox therapeutics	Development of exosomes for niemann-pick and duchenne muscular dystrophy
IBM	A new lab-on-a-chip technology separates smaller biological particles than before
MDimune	Drug delivery
Molecuvax	Cancer
Puretech	New milk exosome oral biologics for biological macromolecules (including antisense oligonucleotides) and complex small molecules
Regeneus	Donor adipose-derived MSCs secrete molecules (including cytokines, growth factors, and exosomes) to reduce pain and inflammation, and promote accelerated healing and repair
Reneuron	Large-scale production of exosomes from immortal stem cell lines
RoosterBio and exopharm	Advanced therapeutic extracellular vesicles/exosomes from adult stem cell sources for clinical practice
Stem cell medicine	MSC exosomes for autism treatment development
Tavec pharmaceuticals	miRNA delivery for cancer treatment
The cell factory	Candidate drug for EVs (CF-MEV-117) is being developed for the treatment of drug-resistant epilepsy in children
Theragnostex	Development of multiple analyses based on early cancer detection of exosomes to determine the best treatments specific to patients
VivaZome therapeutics	Development of advanced manufacturing processes
Ymir genomics	Two high-yield methods for separating EVs and extracellular nucleic acids from urine

EVs, extracellular vesicles; IL-12, interleukin-12; R & D, research and development.

Related reports on the clinical application and practice of exosomes can be found on the website of “clinical trials.” If you use “exosomes” as a keyword for searching, 204 results can be found, including research treatments for treating various diseases. It is notable that most of them are at the recruitment stage of clinical trial volunteers. Exosomes have clinical application prospects, but the lack of a safe and effective delivery system is a problem (Chen et al., 2019b). Although some exosome-related standards are reported, further in-depth research is needed before clinical application (Thery et al., 2018).

## MSC-Exosomes

MSCs are the most scientifically studied cells in regenerative medicine. They play a significant role in tissue regeneration and repair and generate local anti-inflammatory and healing signals (Huang et al., 2017; Wu et al., 2020). Studies have shown that MSCs work through their paracrine effect, namely secreted EVs work. MSC-derived EVs, particularly exosomes, can play an obvious therapeutic role in diseases of tissue damage or inflammation, and they have great potential in the future.

### MSCs

MSCs were originally discovered in bone marrow, and they can also be found in other tissues, such as fat, the umbilical cord, cord blood, the placenta, fetal lung, the amniotic membrane, gingiva, and dental pulp (Zhang et al., 2017; Zhang et al., 2018). MSCs have powerful paracrine function, which is also the main mechanism for their therapeutic effect. They regulate the microenvironment by secreting a series of cytokines, chemical molecules, and growth factors and activate endogenous stem cells to perform tissue repair after damage (Gnecchi et al., 2016).

MSCs have so many advantages, and they are the first choice of seed cells for cell replacement therapy and tissue engineering (Uccelli et al., 2008; Zhao et al., 2019). They have been used to treat different types of disease (Squillaro et al., 2016; Madl et al., 2018; Shi et al., 2018). However, the therapeutic effect of MSCs often fails to meet expectations from the results of a few experiments that have been disclosed. Furthermore, the tumorigenicity of MSCs in transplantation requires more attention. In this way, the preclinical studies of MSCs seem to be unsafe. Therefore, standardized regulations or policies for MSCs urgently need to be formulated. Simultaneously, seeking safer and more effective application schemes are equally important. Therefore, MSC-exosomes are the best alternative choice at this moment.

### Brief Description of MSC-Exosomes

A number of *in vivo* and *in vitro* studies show that MSC-exosomes have the ability to immunoregulate, promote angiogenesis, and regenerate tissue. For example, MSC-exosomes reduce the scope of myocardial injury (Ma et al., 2017); promote tissue damage repair (Zhang et al., 2015), such as acute tubular injury (Bruno et al., 2009), nerve injury (Drommelschmidt et al., 2017) and lung injury (Lee et al., 2012); promote angiogenesis (Merino-Gonzalez et al., 2016); and regulate the immune system (Ti et al., 2015).

### Advantages and Disadvantages of MSC-Exosomes

#### Advantages

##### Advantages Over Stem Cells

Many current studies show that stem cell–derived EVs have similar functions to parent cells. Exosome-based therapies circumvent some of the tricky problems of cell therapy, such as necrosis or abnormal differentiation caused by stress reactions and immune rejection caused by cell transplantation. The main advantages can be summarized as follows. First, exosomes are the mediators of stem cell paracrine action. They participate in the transmission of information between cells and are considered to be the main mechanism of disease treatment. Second, exosomes can be combined with existing, newly developed compositions or methods and designed as carrier particles containing specific ingredients. In addition, they can be engineered to target specific cells or tissues. Third, exosomes have autonomous targeting capabilities and can home to the lesion tissue, which is conducive to constructing them into drug carriers. All these characteristics facilitate exosomes to be the ideal natural material for the development of nanomedicine (Mathieu et al., 2019). Compared with cell therapy, it is safer and has no potential tumorigenicity of stem cells. It is the best alternative to cell-free therapy at present.

##### Advantages Over Synthetic Nanoparticles

In drug delivery, exosomes can not only achieve similar effects to synthetic nanoscale carriers (such as liposomes, nanoparticles), but also have cell-based biological structures and functions. For example, exosomes can provide natural biocompatibility; higher chemical stability; longer distance intercellular communication; and inherent intercellular communication, fusion, and delivery capabilities.

Some studies show that exosomes have the ability to selectively fuse cells and target specific tissues as well as to penetrate tight tissue structures, such as the blood–brain barrier. Liposomes and nanoparticle synthesis systems have a high degree of flexibility in terms of reagent selection, preparation procedures, and surface functionalization. The combination of exosomes and the nanoscale particle synthesis system has greater value in medicine. They can be loaded with more biomimetic materials and nonbiological units.

#### Disadvantages

On the one hand, the lack of criterion may be the main obstacle to clinical transformation. Because the production and contents of MSC-exosomes are closely related to the origin, activity, and neighbors of MSCs, they are dynamic. Therefore, exosomes/EVs derived from MSCs should be studied and applied according to certain specifications.

On the other hand, heterogeneity of MSC-exosomes/EVs is the thorniest problem. It is important to note that it remains difficult to purify a specific EV population, and current EV preparations, including exosomes, are largely heterogeneous. In view of this, the International Society for Extracellular Vesicles (ISEV) recommends the use of the collective term “EVs” unless the biogenesis pathway is demonstrated. In addition, information on the size, biochemical composition, and descriptions of the culture conditions or cell origin should also be provided. Therefore, we face huge challenges in the application of exosomes/EVs, such as the diversity and preparation of MSCs, various methods of EV production and separation, lack of standardized quality assurance assays, and limited reproducibility of *in vitro* and *in vivo* functional assays. To address these issues, members of four societies (SOCRATES, ISEV, ISCT, and ISBT) propose specific coordination standards. Specifically, MSC-derived EVs should be defined by quantifiable metrics to identify the cellular origin of the EVs in a preparation, presence of lipid-membrane vesicles, and the physical and biochemical integrity of the vesicles (Witwer et al., 2019).

### MSC-Exosomes as Nanomedicine

**FIGURE 3:**
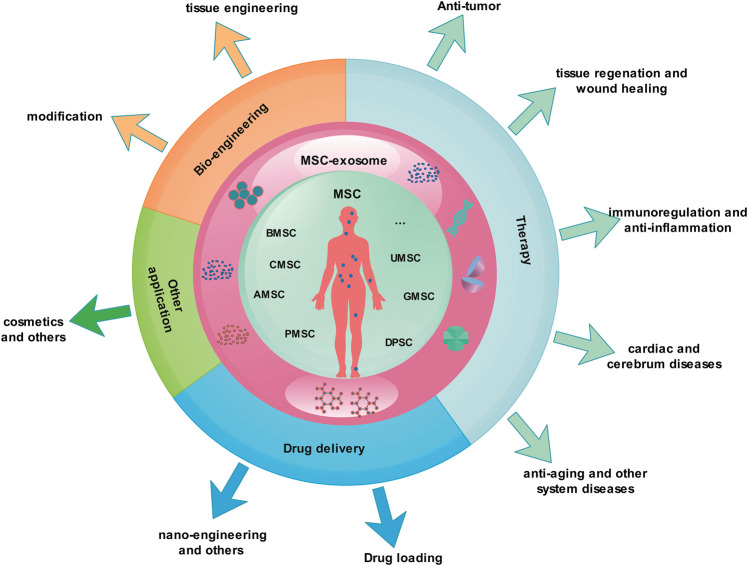
MSC-exosomes as nanomedicine. Most of the MSC-exosome research is in the basic and clinical trial stage and has not yet been formally applied to the clinic. The research covers various fields of medicine and health, including clinical disease treatment, drug delivery, nano-engineering, and bio-engineering. AMSC, Adipose-derived mesenchymal stem cell; BMSC, bone marrow mesenchymal stem cell; CMSC, cord blood mesenchymal stem cell; DPSC, dental pulp stem cell; MSC, mesenchymal stem cell; MSC-exosomes, mesenchymal stem cell-derived exosomes; GMSC, gingival mesenchymal stem cell; PMSC, placenta-derived mesenchymal stem cell; UMSC, umbilical cord mesenchymal stem cell.

MSCs release a large number of exosomes during the culture process, and studies show that the exosomes from them have multiple functions. As nano-scale natural biological particles, exosomes have unique advantages in nanomedicine. They have been researched in many diseases and achieved some results (Figure 3).

#### MSC-Exosomes as Nanocarriers

##### MSC-Exosomes and Functional Micro-RNA Delivery

Utilizing the advantages of MSC-exosomes, therapeutic microRNAs are loaded and targeted to the disease site for therapeutic purposes. A study shows that MSC-exosomes encapsulating microRNA-379 could be targeted to tumor cells (O’Brien et al., 2018). In another study, researchers used MSC-exosomes for nasal administration. These MSC-exosomes loaded with phosphatase and tensin homolog siRNA could finally reach the spinal cord region through the blood—brain barrier, improve symptoms in spinal cord injury model rats, and even restore some function (Guo et al., 2019).

##### MSC-Exosomes and Drug Loading

Drugs or materials can be loaded to play the role of themselves. For example, there have been studies using embryonic stem cell (ESC)—derived EVs to load paclitaxel and artificially endowed certain targeting effects for the treatment of gliomas, and they have achieved success (Zhu et al., 2019). It also has been reported that MSC-derived EVs were coated with hyaluronic acid and played a certain function. This study has enriched our understanding of vesicle components to some extent (Arasu et al., 2017). In chronic liver failure, hydrogel-mediated MSC-exosomes improved liver regeneration (Mardpour et al., 2019). Exosomes derived from bone marrow MSCs (BMSC-exosomes) have been widely used for treating myocardial infarction (MI). However, low retention and short-lived therapeutic effects are still major challenges. Some researchers have used alginate hydrogel for the sustained release of EVs. As a new treatment strategy for MI, it has achieved certain success (Lv et al., 2019).

##### MSC-Exosomes and Engineering

In regenerative medicine, there are many studies based on MSC-exosomes. These medicines combine tissue engineering techniques to achieve tissue repair and regeneration: for example, desktop-stereolithography 3D printing of radially oriented extracellular matrix (ECM)/MSC-exosome bioink for osteochondral defect regeneration. The study suggests that a 3D printed, radially oriented ECM/GelMA/exosome scaffold may be a promising strategy for the treatment of early osteoarthritis (OA) (Chen et al., 2019a). MSC-derived EVs promoted human cartilage regeneration *in vitro* (Vonk et al., 2018).

Research has also focused on improving the production of MSC-exosomes through tissue engineering. For example, there is a study using tangential flow filtration and 3D culture to produce MSC-exosomes. The researchers combined the advantages of the two production strategies to develop a powerful and scalable strategy that is compatible with GMP production of MSC-exosomes. The results show that exosomes produced from 3D cultured MSCs by tangential flow filtration had higher yield and activity (Haraszti et al., 2018).

The advent of nanotechnology has brought new prospects for the treatment of type 2 diabetes (T2DM), including controlled drug release and targeted drug delivery. Therapies based on exosomes have been extensively studied to achieve better drug delivery. Various reports indicate that exosomes can be used as effective nanocarriers to provide small interfering RNAs and microRNAs, proteins, or chemical drugs to improve the treatment of cancer. However, due to the lack of natural targeting ability to pancreatic beta cells, there are few reports of exosomes as drug carriers for the treatment of T2DM (Zhuang et al., 2019). We can combine nanoengineering to start research on the treatment of T2DM with exosomes.

#### MSC-Exosomes as Nanomedicine

##### MSC-Exosomes and Tumor Therapy

On a global scale, malignant tumors threaten human health. The traditional methods, namely surgery, radiotherapy, and chemotherapy, have not yet met clinical needs. Tumor biotherapy is currently attracting attention as a new prospect and is considered the fourth tumor treatment technology. A vast number of studies have highlighted the potential of MSC-exosomes/EVs as new tools in basic research and clinical practice. Some people have been able to prove that MSC-exosomes may affect the level of cytokines, the targeted-delivery of microRNAs, and the status of tumor cells.

For example, EVs derived from human umbilical cord MSCs (UMSC-derived EVs) could promote the migration, invasion, apoptosis, and epithelial—mesenchymal transition (EMT) of lung cancer cell line A549 but inhibit its proliferation. This might be because the UMSC-derived EVs could stimulate the expression of TGF-β, which is the main factor controlling the progress of cancer cells (Zhao et al., 2018b). Another *in vivo* therapy for breast cancer found that engineered MSCs secreted miR-379-rich EVs could support tumor-targeted delivery (O’Brien et al., 2018). Besides this, BMSC-exosomes stimulate circulatory quiescence and dormancy in early breast cancer (Bliss et al., 2016). Subsequently, Casson J et al. report similar beneficial effects (Casson et al., 2018). Moreover, MSC-exosomes are able to enhance the effect of tumor radiotherapy due to their function in tumor growth delay and metastasis control (de Araujo Farias et al., 2018). Although this result is valuable, it is unclear whether MSC-exosomes exert a systemic effect after radiotherapy.

##### MSC-Exosomes and Immunoregulation

Previous studies have reported the immunomodulatory mechanism of MSC-exosomes or EVs (Fierabracci et al., 2015) by regulating immune cells (Harrell et al., 2019), inflammatory cytokines (Luo and Zheng, 2016; Zou et al., 2018; Yang et al., 2019a), and the microenvironment (Shi et al., 2018). These processes are related to both innate and adaptive immune cells, among which the macrophages, CD4^+^Th1, Th17, and Treg cells, and their immunoregulatory miRNAs are crucial.

MSC-exosomes may regulate T cell subsets in autoimmune diseases, which has been reported at the early stage (Blazquez et al., 2014). Currently, in a mouse model with experimental autoimmune encephalomyelitis (EAE), MSC-exosomes reduce the levels of pro-inflammatory Th1 and Th17 cytokines (such as IL-6, IL-12p70, and IL-17AF) but increase the proliferation of anti-inflammatory Treg (Riazifar et al., 2019). Therefore, adipose-derived MSC-derived exosomes (AMSC-exosomes) increase the number of Treg cells and their products without changing the proliferation index of lymphocytes in type 1 diabetes (T1DM) (Nojehdehi et al., 2018). Similar effects are also reported in rheumatoid arthritis (Cosenza et al., 2018) and graft-versus-host disease (GVHD) (Kordelas et al., 2014; Fujii et al., 2018; Lai et al., 2018). Interestingly, Fujii S et al. found that BMSC-derived EVs ameliorated GVHD via suppressing the differentiation of naive T cell populations (Fujii et al., 2018). Macrophages are crucial members of the innate immune response and can be regulated by MSC-exosomes in various diseases (Zhao et al., 2018a; Liu et al., 2019b; Wang et al., 2019c). Depending on the different microenvironment, naive macrophages (M0) can be polarized into pro-inflammatory M1 or anti-inflammatory M2 and play different roles in these conditions (Shapouri-Moghaddam et al., 2018). Wang R et al. demonstrate that gingiva MSC-derived exosomes (GMSC-exosomes) might promote the conversion of M1 into M2 macrophages and reduce the pro-inflammatory factors produced by M1 macrophages (Wang et al., 2019c). Similarly, BMSC- (Liu et al., 2019b) and AMSC-exosomes (Zhao et al., 2018a) obtained consistent results. In addition, BMSC-exosomes also affected the IL-10 production of macrophages during the inflammatory process (Liu et al., 2019b). Moreover, AMSC-exosomes play a role in facilitating metabolic homeostasis of obese mice, thereby attenuating adipose inflammation and obesity (Zhao et al., 2018a).

##### MSC-Exosomes and Wound Healing and Regeneration

For MSCs, we can easily think of their ability to promote damage repair, tissue regeneration, and antiaging; thereby the MSC-exosomes may have similar potential. Indeed, numerous researchers have conducted related studies. First, the role in wound healing of MSC-exosomes or EVs is the most frequently mentioned (Geiger et al., 2015; Zhang et al., 2015; Rani and Ritter, 2016). Early in 2009, Bruno S. et al. proved that MSC-derived microvesicles could protect against acute tubular injury (Bruno et al., 2009). Subsequently, more studies started to be performed. By mediating the interaction between cells and stimulating cell proliferation, MSC-exosomes or EVs can be used for numerous diseases. For example, stem cell—derived exosomes are involved in transferring microRNAs and affecting signal transduction pathways into target cells, which could be used as microRNA therapy for age-related musculoskeletal disorders (Yao et al., 2019). In addition, MSC-derived EVs rescued radiation damage to murine marrow hematopoietic cells through stimulating proliferation of the normal murine marrow stem cell/progenitors (Wen et al., 2016). Moreover, GMSC-exosomes can promote skin wound healing (Shi et al., 2017b) and nerve regeneration (Rao et al., 2019). However, further research is required to explore the molecule mechanism and signal pathway. La Greca A et al. analyze the proteomic of MSC-exosomes and attempts to find the differences that might confer MSC-exosomes’ therapeutic properties (La Greca et al., 2018). In view of the current immature understanding of MSC-exosomes, the specificity of MSC-exosomes and cell-to-cell communication need more in-depth discussion (Mathieu et al., 2019).

Simultaneously, current evidence suggests that MSC-exosomes or EVs exert effects by activating related receptors and pathways. For example, MSC-derived EVs activate VEGF receptors and vital pro-angiogenic pathways (SRC, AKT, and ERK), resulting in accelerating recovery of hind limb ischemia (Gangadaran et al., 2017). UMSC-exosomes activate Wnt/β-catenin signaling to enhance the stemness of UMSCs, thereby promoting wound healing (Shi et al., 2017a). As mentioned earlier, the microRNAs in MSC-exosomes or EVs play important role in tissue regeneration and age-related diseases (Yang et al., 2019b; Ullah et al., 2019). Ratajczak et al. first reported that ESC-derived EVs supported self-renewal of hematopoietic progenitors and multipotency by transfer of growth factors and mRNAs, which might modulate the phenotype of target cells (Ratajczak et al., 2006). Recently, studies show that BMSC-exosomes alleviate osteoporosis by mediating the microRNA-34c/SATB2 axis (Yang et al., 2019b). Likewise, more recent work reports that MSC-derived EVs—microRNAs may be involved in various processes leading to aging, including cellular senescence, exhaustion, telomere length, and circadian rhythm (Ullah et al., 2019). Of course, the mechanisms of MSC-exosomes or EVs may be diverse, such as proteins, lipids, etc. (Tofino-Vian et al., 2018; Liao et al., 2019; Zhang et al., 2019), which are worthy of further study.

##### MSC-Exosomes and Nervous System, Cardiac, and Cerebrum Diseases

Early work show that neural stem cell—derived EVs have anti-inflammatory, neurogenic and neurotrophic effects, which may be expected to be used to treat a variety of neurodegenerative diseases (Vogel et al., 2018). Furthermore, MSC-exosomes contain multiple neuroprotective proteins, which may be used as cell-free therapies to treat central nervous system disorders (Riazifar et al., 2019). Also, combined with the results of *in vivo* and *in vitro* experiments, it is proven that MSC-exosomes ameliorate inflammation-induced astrocyte alterations via affecting the Nrf2-NF-κB signaling pathway, resulting in attenuating reactive astrogliosis and inflammatory responses, ameliorating learning and memory impairments in mice (Xian et al., 2019). Additionally, in the brain of AD mice, after hypoxic treatment with MSCs, the level of miR-21 from MSC-exosomes increased effectively, which could restore the cognitive deficits and prevented pathologic features (Cui et al., 2018). These studies provide good examples of MSC-exosomes that can be regarded as nanotherapeutic agents. After these studies show the therapeutic potential of MSC-exosomes or EVs in neurodegenerative diseases, the focus of this field is on cardiac and cerebrum diseases. Early studies show that MSC-exosomes or EVs could reduce myocardial ischemia/reperfusion injury (Lai et al., 2010) and might lead to recovery after stroke (Kim et al., 2012). Moreover, recent research demonstrates that UMSC-exosomes and ESC-exosomes were found to be effective in MI (Khan et al., 2015; Shao et al., 2019). Much more interestingly, the contractility and stroke of human cardiac tissue could also be regulated by microRNAs from MSC-exosomes or EVs (Mayourian et al., 2018; Moon et al., 2019).

##### MSC-Exosomes and Other Diseases

Perhaps considering that MSC-exosomes or EVs may potentially function as nanotherapeutic agents for various disorders, numerous current studies on MSC-exosomes or EVs may provide crucial clinical evidence. MSC-exosomes or EVs are related to diseases of all human systems. In addition to the aforementioned studies, other system or tissue disorders have been studied as well, such as those related to lung (Schoefinius et al., 2017; Dong et al., 2018; Mansouri et al., 2019), liver (Liu et al., 2018), muscle (Bier et al., 2018; Wang et al., 2019a), and pancreas (Sun et al., 2018; Fan et al., 2019a). For example, MSC-exosomes improved peripheral neuropathy in a diabetic mouse model (Fan et al., 2019a). Moreover, UMSC-derived EVs could reverse peripheral insulin resistance and relieve β-cell destruction to alleviate T2DM (Sun et al., 2018). AMSC-exosomes alleviated acute liver failure (Liu et al., 2018) and prevented the muscle degeneration associated with torn rotator cuffs (Wang et al., 2019a). Besides this, experimental pulmonary fibrosis (Mansouri et al., 2019), lung adenocarcinoma (Dong et al., 2018), and cell survival (Schoefinius et al., 2017) may be influenced by MSC-exosomes or EVs.

#### MSC-Exosomes and Cosmetics

In 2018, ExoCoBio, a global biochemical start-up company in the field of exosomes, launched ASCE+, a mesotherapy medical beauty brand that can reverse the skin clock. It is a product made by freeze-drying the exosomes (ASC-EXOSOME™) derived from a stem cell culture medium. It is the first biological product in the world to activate its own adipose tissue. This large-scale investment will become the cornerstone of the development of exosome-based cosmeceuticals and biopharmaceuticals.

### Clinical Application and Commercial Production of MSC-Exosomes

A large number of studies show that EVs released by stem cells have repair functions similar to stem cells. Therefore, many preclinical studies have begun to turn to stem cell—derived EVs to repair body damage. Research on the production and testing of clinical-grade exosomes is meaningful. Currently, a study reports a method for large-scale production of exosomes by BMSC in accordance with GMP standards, which provides detailed procedures and the obstacles needed to be overcome in the production of clinical therapeutic exosomes. In this study, we mention several standards for mass production and quality control of exosomes permitted by GMP, including the factors of determining the size distribution of exosomal preparations, analysis of exosomal markers, and determination of potency (Mayourian et al., 2018). This research has laid a good foundation for the clinical application of exosomes (Mendt et al., 2018). Exosomes secreted by stem cells are a new type of therapeutic products very suitable for regenerative medicine applications, but the bottlenecks in large-scale stem cell production prevents the widespread clinical application of these natural bioactive nanoparticles.

### Development Prospects and Research Models

**FIGURE 4:**
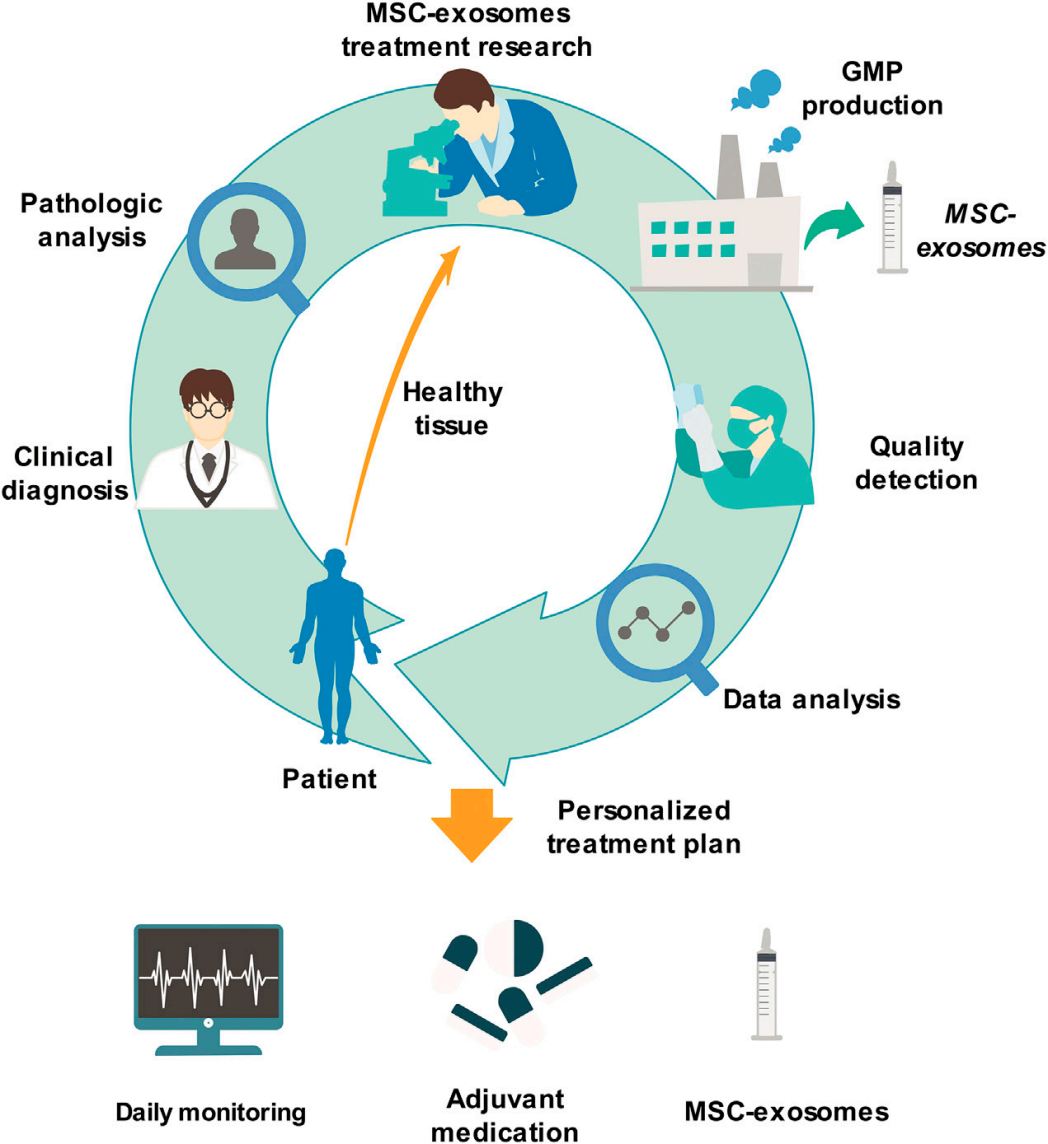
Personal therapeutic scheme of MSC-exosomes. Through clinical diagnosis and analysis of patients, we can extract MSCs from patients' healthy tissues, culture them *in vitro*, prepare MSC-exosomes according to the standardized requirements of GMP, and formulate private treatment plans for patients after strict quality inspection. During the treatment period, measures, such as daily monitoring of disease indicators and adjuvant medication, are finally provided to achieve personalized and precise treatment. GMP, Good Manufacturing Practice; MSC-exosomes, mesenchymal stem cell-derived exosomes.

Because MSC-exosomes have many advantages, they will certainly make great progress in the medical domain in the future. More energy should be devoted to basic research on MSC-exosomes, such as further exploring their treatment mechanism, effective extraction and separation methods, and standardized production. Simultaneously, clinical research also needs to be actively performed by comprehensively considering various factors related to patients’ life security. The clinical application of MSC-exosomes will make great progress in the field of personalized and precision treatment. At that time, through clinical diagnosis and analysis of patients, we can extract MSCs from patients' healthy tissues, conduct *in vitro* culture, prepare MSC-exosomes according to the requirements of GMP standards, and formulate private treatment plans for patients after undergoing strict quality inspection. During the treatment period, measures, such as daily monitoring of disease indicators and adjuvant drugs, are provided to ultimately achieve personalized and precise treatment (Figure 4).

## Conclusion and Outlook

In recent years, progress on exosomes has begun to enter the field of application. As a branch of liquid biopsy, exosomes have brought new imagination to the diagnostic market. As a potential nanomedicine, MSC-exosomes show unique advantages in disease treatment and drug delivery. They not only play a role in the treatment of diseases, such as tumors, immunity, the nervous system, and regenerative medicine, but they also provide a basis for the diagnosis and prevention of diseases. As a natural biological nano-carrier for drug delivery, its advantages are also very prominent. It combines with multifield engineering to help medical development. With the development of personalized and precision medicine strategies, exosomes are bound to occupy a place in the future of the medical field.

Although the study of MSC-exosomes has achieved a lot of promising results, in general, our exploration in the field of exosomes is still in its infancy. There is still a long way to go in the future. At present, there are still some problems to which we need to pay attention. For example, the methods of exosomal isolation and purification are not uniform, which affects the reproducibility of research results. The isolation method of body fluid exosomes is still immature. The fate of exosomes after entering the recipient cells, the specificity of organ distribution, and the treatment mechanism of disease are still unclear. In addition to these obstacles, low yield (Shao et al., 2018), heterogeneity (Pegtel and Gould, 2019), difficulty preserving (Jeyaram and Jay, 2017), and so on need to be solved urgently. It is difficult to achieve standardized, large-scale production. In order to produce standardized exosomes for clinical use, it is necessary to determine the performance characteristics of exosomes, including authenticity, precision, clinical sensitivity and specificity, linearity, analytical sensitivity, analytical specificity, sample stability and diversity, and uncertainty of measurement (Ayers et al., 2019).

We need to accept the uncertainty of MSC-exosomes and that no theory is a universal concept that can be applied to every disease and to pay close attention to preclinical outcomes and their relevance to future clinical studies. With further research, we believe that these problems will be gradually resolved. We have reason to believe that exosomes are important “bridges” for cell-to-cell communication and will have a broader space for development in the future.

## Glossary

AD: Alzheimer's disease
Alix: apoptotic junction gene 2 interacting protein X
AMSC: adipose-derived mesenchymal stem cells
BMSC: bone marrow mesenchymal stem cells
CDCs: Capricor's cardiosphere-derived cells
CHO: Chinese hamster ovary cells
CMSC: cord blood mesenchymal stem cells
DPSC: dental pulp stem cells
EAE: experimental autoimmune encephalomyelitis
ESCRT: endocytosis sorting transport complex
EVs: extracellular vesicles
FDA: food and drug administration
GFP: green fluorescent protein
GMP: good manufacturing practice
GMSC: gingival mesenchymal stem cells
GVHD: graft-versus-host disease
ILVs: intraluminal vesicles
ISCT: the International Cell Therapy Association
MI: myocardial infarction
MSC-derived EVs: mesenchymal stem cell derived EVs
MSC-exosomes: mesenchymal stem cell-derived exosomes
MSCs: mesenchymal stem cells
MVBs: multivesicular bodies
NSCEVs: neural stem cell-derived extracellular vesicles
NSCLC: non-small cell lung cancer
OA: osteoarthritis
PMSC: Placenta-derived mesenchymal stem cells
T1DM: type 1 diabetes
T2DM: type 2 diabetes
TSG101: tumor susceptibility gene 101 protein
UMSC: umbilical cord mesenchymal stem cells
